# Biological and cognitive theories explaining panic disorder: A narrative review

**DOI:** 10.3389/fpsyt.2023.957515

**Published:** 2023-01-30

**Authors:** Peter Kyriakoulis, Michael Kyrios

**Affiliations:** ^1^Faculty of Arts, Health and Design, Swinburne University, Hawthorn, VIC, Australia; ^2^College of Education, Psychology and Social Work, Órama Institute for Mental Health and Wellbeing, Flinders University, Bedford Park, SA, Australia

**Keywords:** panic disorder, etiology, biological theories, psychological theories, narrative review

## Abstract

The current narrative review summarizes and examines several theories of panic disorder (PD) including biological theories, encompassing neurochemical factors, metabolic and genetic theories, respiratory and hyperventilation theories and cognitive theory. Biological theories have informed the development of psychopharmacological treatments; however, they may be limited in their utility given the efficacy of psychological treatments. In particular, behavioral and, more recently, cognitive models have garnered support due to the efficacy of cognitive-behavior therapy (CBT) in treating PD. The role of combination treatments has been found to be superior in the treatment of PD in particular cases, lending support for the need for an integrated approach and model for PD given that the etiology of PD is complex and multifactorial.

## Introduction

Panic disorder (PD) is an anxiety disorder characterized by spontaneous and recurrent panic attacks (PAs) which is severe and persistent, as specified by *The Diagnostic and Statistical Manual of Mental Disorders* 5th ed. (DSM-5) (1). PAs are characterized by multiple physical symptoms including a pounding or racing heart, sweating, dyspnea, weakness or dizziness, feeling hot or cold chills, tingling or numbness in the hands, chest pain, or stomach pain (1). PD has often been treated by either psychopharmacological interventions and cognitive behavioral therapy (CBT) or a combination (2).

This paper reviews theories describing the etiology of PD which have relevance for our understanding of this common debilitating disorder and its treatment. The last review on theories explaining panic disorder was published over 10 years ago (3) and, while effective treatments for PD exist (4), there remains room for improvement (5) in outcomes. This paper undertakes a narrative review examining and summarizing the major approaches to understanding PD and frameworks for its treatment. Biological theories of PD have been proposed, encompassing neurochemical factors, metabolic and genetic theories, respiratory and hyperventilation theories. Furthermore, five major psychological theories have been described as bases for understanding the etiology of PD, including conditioning and related behavioral theories, cognitive theories, anxiety sensitivity theory, and psychodynamic theories. Of these, due to their current widespread use clinically, this paper focuses on cognitive and cognitive-behavioral theories of PD (6, 7).

Despite both biological and psychological frameworks offering treatment avenues for PD, to date, they remain largely unintegrated (8). There is as yet little agreement of how best to integrate both models nor how each of the frameworks could evolve treatment approaches by integrating emerging research trends. Such integrative activity gives the potential to develop new, combined or augmented treatments.

## Biological theories

### Neurochemical theory

A neurochemical imbalance of neurotransmitters in the brain, such as serotonin, norepinephrine, dopamine, and gamma-aminobutyric acid (GABA), is thought to cause PD symptoms (9). This biological theory is evidenced by the symptom reduction effects of antidepressant or anxiolytic medication in many PD suffers (10). Treatments of PD have utilized several groups of drugs including; benzodiazepines, tricyclic and heterocyclic antidepressants, monoamine oxidase inhibitors (MAOIs) and selective serotonin reuptake inhibitors (SSRIs) (11). Nowadays, antidepressants used for aminergic symptoms are more commonly used to treat panic symptoms rather than ones that target the principal inhibitory neurotransmitters in the central nervous system (CNS) such as benzodiazepines that work on the GABA receptors (12). However, it is widely accepted that despite their effectiveness, medications that target the GABA receptors come with a high risk of addiction and impact on cognitive functioning (13).

Neurochemical theories further propose dysregulated functioning of neurochemicals leading to a deficiency in the serotonergic system or excess serotonin, in an attempt to explain the etiology of PD (14). Additionally, abnormal chemoreceptor reactivity may be involved in the etiology of PD. Amongst some of the inhibitory and excitatory neurotransmitters that may play a role in the upregulation or downregulation of panic may include glutamate, GABA, cholecystokinin, adenosine, dopamine, and norepinephrine (15). The inhibitory/excitatory framework has also been influential in recent behavioral approaches to treatment (16), and will be discussed in a following section.

Other neurochemical theories have suggested the significant involvement of the neuropeptide Y (NPY), given its a dense concentration in anxiety circuits, which has been thought to be involved in the consolidation of fear memories (17). Evidence suggests that NPY contains anxiolytic properties and serves the role as a protective neurochemical that facilitates stress resilience (18). NPY is saturated in the central nervous system, particularly in the cortical, limbic, and hypothalamic (i.e., basal ganglia, hippocampus, hypothalamus, amygdala, nucleus accumbens, cortex, periaqueductal gray, and lower brainstem) brain region (19) where located and plays a vital role in energy homeostasis, pain, control of food intake and physiological processes related to stress/stress resilience (20). Patients with anxiety disorders have showed reduced concentrations of NPY, therefore, they experience elevated risk to stress and fear responses.

### Neuroanatomical theories and animal models

An association of brain regions being controlled by an anxiety network have been described by numerous researchers who have examined fear circuits in the brain (21–23). The thalamus is a relay station for sensory input and receives information from all senses, and analyses bodily inputs that are threatening (24). The thalamus then initiates two strategies; one is a fast and general emergency response whilst the other one is a slower, detailed analysis of the feared situation.

In the initial emergency response, the thalamus analyses the situation to determine whether the presenting threat corresponds to an innate instinctual fear. It does this by comparing the fear situation with primitive fears that are uniquely stored in an inborn survival memory (17). In an effort to maximize chances for survival in a life-threatening situation, the amygdala is alerted and a number of responses are initiated, including the activation of the sympathetic nervous system and the HPA axis.

In the slow response, the hippocampus, which is associated with learning and memory analyses the threatening situation by retrieving detailed information from all available memory sources; this includes gathered information over the years including theoretical knowledge, factual information and the recall of past unpleasant experiences. This ability assists the hippocampus to develop context and assess the situation more realistically. The amygdala is then informed that the threatening situation is not dangerous, down regulating the fear response, and consequently scaling down the physiological response.

In recent years, animal studies and brain imagery techniques have been used extensively to identify brain regions which are specifically implicated in the regulation of panic and anxiety. The limbic area, particularly the amygdala has been directly associated with the fight/flight response (25). Moreover the locus ceruleus (LC) appears to be implicated in the sleep-wake cycle, arousal, anxiety, and fear which were commonly identified brain regions relating to PD (26, 27).

Neuroimaging studies have also highlighted the characteristic of hypoactivation in the prefrontal cortex (PFC) is observed in anxiety disorders that involve intense fear such as panic, social anxiety disorder, and post-traumatic stress disorder (PTSD), which in turn decreases the amygdala inhibition (28). In contrast, anxiety disorders that involve rumination and obsessiveness such as Generalized Anxiety Disorder (GAD) and Obsessive Compulsive Disorder (OCD) are characterized by overactivity in the PFC (28). Another neuroanatomical area thought to be involved in PD is the dorsal raphe nucleus (DRN). The DRN subdivisions comprise distinct populations of neurons that vary in their morphological nature, neurochemically and functionally. It has been found that anxiety and panic activate different subset of neurons in the DRN (29–31).

A study conducted by Johnson and colleagues found that exposing rats to high concentrations of hypercarbic gas substantially increased anxiety and elicited components of an adaptive panic/defense –related response as observed by increases in autonomic arousal (32–34). Disrupted GABA inhibition in the dorsomedial hypothalamic perifornical region (DMH/Pef) resulted in rats being susceptible to the induction of PAs following sodium lactate infusions (35). The researchers speculated that the DMH/Pef regions mediate the respiratory and autonomic, behavioral and endocrine components of the panic like response, which includes elevated heart and respiration rate, increased blood pressure, plasma catecholamine, intestinal and colonic mobility. Moreover, on the basis of animal research, there has been increased interest in the use of augmenting agents such as d-cycloserine to enhance response to cognitive-behavior therapy and, especially, exposure therapy for PD. However, despite initial augmentation of efficacy, longer-term impacts have been less successful (36). While multiple treatment studies have examined the effectiveness of pharmacological agents, few studies have focussed on neuromodulatory treatments for PD. A relatively recent review found only one of two randomized trials demonstrating efficacy of transcranial magnetic stimulation (TMS) for PD (37). A previous Cochrane Review had also concluded that there was insufficient evidence supporting the use of repetitive TMS for the treatment of PD (38). There are no studies on the use of electroconvulsive therapy for a primary diagnosis of PD (39). Hence, there is still much work to be conducted in the integration of brain-based models of PD into treatment frameworks.

### Hormone theory

It has also been well documented that hormones, particularly gonadal hormones, play an influential role in influencing the frequency and intensity of PAs. Spontaneous PAs affect more women than men, and rarely start before puberty or after menopause, suggesting reproductive hormones may play a significant part in the occurrence of PAs in women (40). Harvard Medical School conducted a national comorbidity survey and found the lifetime prevalence of PD in the U.S. population stands at 4.7%, overall, 6.2% comprised of women, posing more than twice as likely than men at 3.1% (41). Interestingly, women who suffer from PD displayed decreased panic symptoms during pregnancy, delivery, and lactation, although an exacerbation of panic symptoms was observed in the post lactation phase (40). These changes are believed to be characterized by increased levels of progesterone, estrogen, and oxytocin during pregnancy and lactation (42). This implies that some pregnancy hormones may act as a protective factor toward PAs, given the less frequent and intense PAs during this period despite the pregnancy and childbirth period being thought of as one with marked and heightened anxiety and common catastrophic interpretation of physiological changes (42). However, some authors question the specific clinical relevance of high hormonal levels to PD. For instance, Masdrakis and colleague concluded that, in a sample of 24 consecutive acutely-unwell, medication-free PD patients, oxytocin plasma levels were more likely relevant to the severity of their general anxiety symptoms, rather than their specific panic symptoms (43).

### Metabolic theories

Metabolic theories have also been quite popular suggesting that PD sufferers are more reactive to certain anxiety-inducing substances or conditions such as injections of lactic acid, elevated CO_2_ levels, caffeine, yohimbine, m-cholorophenylpiperazine, cholecystokinin (CCK), nicotine, and alcohol (44). Researchers have used numerous procedures to try to reproduce panic-like symptoms in order to fully understand the etiology of PD, suggesting further evidence for the significance of biological theories. Some of the earlier chemicals used to successfully trigger panic like symptoms included epinephrine and norepinephrine (45–47). Among the most common procedures used include the pharmacological agent sodium lactate (48–51), hyperventilation and CO_2_ challenges (52–54).

Researchers postulated an implication of the adenosine system in explaining panic and anxiety since caffeine challenge tests have been known to induce panic like symptoms (55, 56). Adenosine receptors (ARs) are located and dispersed in all brain areas, and are responsible for regulating the release of neurotransmitters and the action of neuromodulators which include both inhibitory and excitatory functions (57).

The modulation of anxiety in the hypothalamus, pituitary gland, especially anterior pituitary gland has also been highlighted as important areas, these areas are involved in the synthesis and release of a number of stress-related hormones (58). A breakthrough in further understanding panic was made in the 1980s, when clinical panic was distinguished from stress-like reactions and common fear based on the finding that clinical and lactate-induced panic did not activate the hypothalamus-pituitary adrenal (HPA) axis (59). The HPA axis and its activity has been extensively researched predominantly in patients suffering from psychiatric illnesses, namely depression and anxiety disorders (60). Results to date have been inconsistent, however, studies reveal that HPA axis dysregulation is common in PD patients (60).

Behavioral responses that characterize PD patients are largely due to the hypersensitivity to CO_2_ in the following three brainstem areas: the peri-aqueductal gray (PAG), the raphe nuclei (RN), and LC (61). The LC is a small nucleus located in the brain stem which contains 70% of all noradrenergic neurons in the brain. When stress activates the LC, it results in increased norepinephrine release in projection sites of the LC, including the amygdala, PFC and hippocampus (62). Serotonin-producing cells in the RN have also been identified as CO_2_ sensors and play a significant role in detecting CO_2_ (63). These behave as pH regulators as they regulate pH homeostasis and have been proposed to form the cellular link between serotonin, chemoreception, and panic.

The PAG has also been implicated in the pathogenesis of panic. This is evidenced by awake patients who have undergone neurosurgery involving the stimulation of the PAG. These patients elicited remarkably similar symptoms to those reported by PD patients when enduring a PA (64). These findings support the notion of a continuous trait, based on one's individual sensitivity response to increasing concentrations of CO_2_ (60). Panic patients are considered to have high sensitivity to rising CO_2_ levels, whereas individuals suffering from congenital central hypoventilation syndrome (CCHS) and divers have low sensitivity to CO_2_ levels rising (65, 66).

### Genetic theories

Amongst the most prominent biological theories are the genetic and hereditary studies that proposed individuals with PD may have inherited genetic predisposition toward the disorder. The prevalence of PD among families of PD patients is very common with evidence suggesting that amongst first-degree relatives of PD patients, there have been up to threefold increases in prevalence. Moreover, it was observed that 25% of the first-degree relatives of PD patients received a diagnosis of PD amongst first-degree relatives of PD patients (67–69).

Twin studies have contributed valuable information including that 31% of the monozygotic twins had a similar diagnosis compared to 0% of dizygotic twins (70). Another study showed that PAs in monozygotic twins were five times as likely to occur than in dizygotic twins PD patients. However, this study only used a small number of twins and the results may not be conclusive and may be explained by the fact that monozygotic twins share more similar environmental experiences and are commonly treated more similarly than dizygotic twins.

Other twin studies have proposed a notable hereditary correlation contribution of 30–48% for PD and > 50% for agoraphobia with the pathogenesis of PD (71, 72). Criticisms of genetic theories have included failure to yet identify a mode of inheritance in line with Mendelian patterns, thus pointing to a complex genetic inheritance model with interactions of multiple vulnerabilities and genes (17). To date, only a few risk genes have been identified and even less explored is the gene-environment interactions specific to PD. Moreover, treatment or intervention implications have yet to evolve but approaches to individualized medicine are beginning to discuss the potential role of pharmacogenetics.

Recent developments in pharmacogenetics, although limited and at its infancy, appear to offer noteworthy information regarding the individuality factor or lack thereof of the response of PD associated genes toward medication (73). It is commonly argued that SSRIs is the recommended first-line psychopharmacological intervention in PD (74). Associations between PD and the serotonin system were observable through 5-HT manipulation (74), a link between SLC6A4 gene polymorphism may predispose individuals to PD (75), and an increased susceptibility for PD for 5-HT2A/5-HT1A serotonin receptor genes (76). Interestingly, certain reviews have pointed out inconsistency within the standard pharmacological treatment of different cultural population (i.e., Asian, Caucasian), which calls for a greater focus on individualized pharmacogenetics (73).

### Respiratory and hyperventilation theories

Neurobiological theories namely the respiratory and hyperventilation theories attempt to explain the etiology of panic based on the notion that PAs may be caused by a dysfunctional respiratory system (53, 77–80). Several theorists have proposed a causality relationship between hyperventilation and PAs, particularly characterized with an imbalance between O_2_ inhaled and CO_2_ exhaled during hyperventilation, thus reducing CO_2_ levels in the body (81). Individuals who hyperventilate in an effort to compensate for the reduction in respiratory rate experience secondary systems, which include shortness of breath, dizziness, trembling, and palpitations (72, 82).

The abovementioned findings led Klein to propose that the principal disturbance in PD may be explained by dysfunctional suffocation monitor (a “false suffocation alarm”, FSA), with dyspnea as the primary feature (42). The FSA asserts that high CO_2_ levels typically serve as a warning marker that the individual may suffer from imminent suffocation, given that high levels of CO_2_ correspond with low levels of O_2_. According to Klein, this suffocation threshold is pathologically lowered in PD patients where the FSA is argued to be hypersensitive to CO_2_ compared to non-PD sufferers, with low levels of CO_2_ becoming a signal for low O_2_ supply. As a result, the brain's suffocation monitor is incorrectly activated based on the signal lack of O_2_ and misfires, therefore, triggering an FSA. Klein hypothesizes that since PD patients believe they are suffocating, they begin to experience PA symptoms such as: shortness of breath, and hyperventilation in order to keep CO_2_ levels well below the suffocation threshold. This implies that hyperventilation is a consequence and acts as a defense response against panic onset instead of a cause of PA. Breathing mechanisms are considered to operate base on a widespread neutral network which involves the medulla, hypothalamus, limbic area, and cortex.

Dysfunction in the endogenous opioid system has a possible etiological mechanism in PD and was proposed by Preter and Klein (78). In particular, it has been suggested that such dysfunction is mechanistically associated with decreases in the threshold of the suffocation alarm. This hypothesis was supported by the findings of Preter and colleague, who demonstrated that lactate infusions in naloxone-treated healthy participants produced feelings and symptoms which mimic those of PAs (79).

Research by Preter and Klein (83) conclusively showed a physiological link between panic-like suffocation in healthy adults and endogenous opioid system deficiency. According to Preter and Klein, episodic dysfunction of the opioidergic systems leads to a decreased threshold of the suffocation alarm, thereby producing PAs (25, 84). Graeff proposed that endorphins increase suffocation sensitivity and separation anxiety in sufferers of PD increasing their vulnerability to experiencing PAs (14). The internal homeostatic milieu comprised of tight parameters involving pH homeostasis and chemosensation remain an important area of study that furthers our understanding of the pathophysiology and treatment of panic disorder. PAs have been conceptualized as resulting from dysfunction of 5-HT inhibitory projections to dorsal regions of DPAG that detect and process threat that is within close proximity, innate fear, or hypoxia. A faulty interaction between serotonin and opioid receptors in the DPAG has been associated with the onset of PAs (14). Hypoxia can be considered as an acute interoceptive threat activating a panic attack as circa strike defense which involves fight, flight, or freezing responses (31).

The ventral respiratory nuclei in the medulla plays a significant role in respiratory drive, which control the phrenic nerve activity as well as the sense of pH and CO_2_ levels in the cerebrospinal fluid (85, 86). Schenberg postulates that the SFA hypothesis presumes that the gas sensors that sense pH levels and CO_2_ levels changes, and the suffocation alarm system may be utilizing different neural pathways (14, 25).

A number of studies have highlighted PD patients experiencing a dysregulation of respiratory physiology (87–89). Respiratory abnormalities are noted in particular in participants with PD as compared to healthy controls and other anxiety disorders (90). In this study, the comparison between groups showed that PD differed with Social Phobia and Generalized Anxiety Disorder, where they demonstrated at baseline, lower end-tidal CO_2_ pressure and a higher mean respiration rate. Furthermore, PD group exhibit lower venous CO_2_ pressure and higher in the bicarbonate ion concentrations compared to other anxiety groups. Such unique baseline respiratory abnormalities and the chronic hyperventilation have been characterized to be specific of PD pathophysiology when compared to other anxiety disorders (91).

PD commonly co-occurs in patients with respiratory abnormalities (91). Indeed, the lifetime prevalence of respiratory disorders including chronic obstructive pulmonary disease (COPD) and asthma among patients suffering from PD is estimated to be 47% (92). There has been suggestion that the central part of PD is the respiratory dysfunction according to reports of high comorbidity between PD and respiratory abnormalities, (93). Moreover, research findings speculate that there may be a familial link between PD and COPD (94). A study suggests that 37 % of primary care outpatients suffering from COPD with depressive symptoms also had comorbidity of anxiety symptoms as well (95). Hyperventilation has been known to activate symptoms such as numbness, tingling sensations, dizziness, and muscle hypertonicity. These symptoms are consistent to hypocapnia and respiratory alkalosis, which results from an increased gas exchange in the lungs (96).

In particular, ample theoretical and empirical evidence links hyperventilation to anxiety and panic. Low CO_2_ levels can occur even in the absence of functional breathing disorders due to the highly soluble nature of CO_2_ (being twenty times more soluble than O_2_). Stress, anxiety, and panic states can cause hyperventilation which leads to the depletion of CO_2_ (97) and results in reduced cerebral blood flow and increased neuronal excitability (82). Intracellular pH and cellular metabolism and the regulation of cerebro-spinal fluid pressure can be disrupted or impaired (78). Hypocapnia produces bronchoconstriction in the lungs and vasoconstriction in the blood vessels (78). Adverse effects can also result in blood pressure, myocardial contractility, cardiac blood flow, pH regulation and electrolyte balance. Unsurprisingly, the association between breathing dysfunction and hyperventilation was established given the hyperventilation theories and evidenced by the extensive physiological effects of hypocapnia and the consequences of respiratory alkalosis, (98) supports to explain panic.

The respiratory subtype of PD, was identified by Briggs, Stretch, and Brandon (1993) and was characterized by prominent respiratory symptoms in PD patients. The prevalence rate of dysfunctional breathing in the general population has been as high as 5–11% (99–103). In asthma sufferers, it as high as 30% (104) and in anxiety sufferers as high as 83% (76). Consistent with previous findings, respiratory subtype has been correlated with high CO_2_ sensitivity (105–108). Furthermore, an altered level of CO_2_ sensitivity in the respiratory PD subtype has been identified in response to panic provocation challenges including the CO_2_ challenge and breath-holding (108–110). Respiratory abnormality in PD is extensive and widely supported by research and propose that PAs result from respiratory abnormalities instead of fear and anxiety alone (30, 111–113).

### Summary of biological theories

In summary, the biological theories have provided well-grounded comprehension into discerning the pathophysiology and etiology of PD. Whilst biological theories have informed psychopharmacological treatments that have been successful in treating panic attacks (114, 115), there are a number of emerging trends from the biological area. The empirical and clinical evidence suggests that first-line psychopharmacological treatments for PD include SSRI's and benzodiazepines. The former is best accompanied by CBT and the latter used short-term to manage PD symptoms (116). Dubovsky and Marshall in their review corroborates evidence contrary to the trend in prescribing patterns favoring SSRI's over BZ in anxiety treatment (116). Nardi and Quagliato suggest the need to reassess the use of BZ in long term treatment of panic vs. short term, as a number of patients may benefit from BZ as first-line treatment (117). A recent review evaluating therapeutic effects of repetitive transcranial magnetic stimulation (rTMS) did not confirm the therapeutic potential for PD (118).

Furthermore, biological theories have been limited in terms of their utility in the development of treatments outside of psychopharmacology. In terms of efficacy, cognitive theories, particularly driven and informed by the development of Cognitive Behavioral Therapy (CBT), have gained greater acceptance as being better in treating and managing PD (119, 120), which appears to be the preferred choice for a first line treatment. This is evidenced by participants in studies without a control condition despite being on a therapeutic dose of medication, have demonstrated improvement following the introduction of CBT (121, 122). Several treatment studies have also shown that both short term and long-term behavioral therapy which aim to reduce one's adverse response to interoceptive and exteroceptive triggers may be equally effective as cognitive therapy in reducing panic disorder (123, 124).

### Cognitive theories

The cognitive-behavioral model of panic disorder identifies key factors that maintain panic disorder (120). The latest composite model of panic adopted several theories into one to explain causality (3). Earlier cognitive theory by Clark suggests that development and maintenance of PD is proposed to begin from the first PA, which most often occurs during a time of stress and is accompanied by “catastrophic misinterpretations” of somatic and other sensations as key (121–124). Irrespective of the source of an individual's internal physical sensations, an individual could develop panic, which in turn leads to catastrophic thoughts about their impending doom, such as “I am going to die”, or “I am having a heart attack”. The catastrophic thoughts sustain the vicious cycle, where anxiety provoking thoughts produce further somatic symptoms and continues the cycle of catastrophic thinking, thus inevitably ends in a PA. Internal focus on somatic and physical sensations has been linked to persistent vigilance and hypersensitivity to normal and common symptoms (125).

An element of self-efficacy was added into the vicious cycle where it is argued that an individual's negative perception of their coping abilities against threat also acts as a perpetuating factor to PA (3). The self-efficacy was perceived as parallel yet simultaneous in sustaining the vicious cycle. According to this particular cognitive model, one's low self-esteem results in high arousal which begins the panic cycle.

Cognitive and personality factors have been implicated (126–128) to place individuals at risk for the vigilance and misappraisals. In particular, “anxiety sensitivity” has been touted as one such vulnerability factor (129). The success of cognitive therapy PD treatment provides support for the misappraisal theory (e.g., Clark et al.). Further evidence has been demonstrated whereby hypothetical stimulations of catastrophic thinking, for instance by reading paired word combinations related to bodily sensations and catastrophes (e.g., “palpitations – die” and “breathless – suffocate”) has been found to increase the likelihood of panic attacks (122).

On the other hand, reduced catastrophic cognitions lead to a reduced probability of panic as a response to panic provocation as well (123). Whilst support for cognitive explanations of PD is present, Bouton and colleague notes two problems with regard to this approach (125). Catastrophic cognitions are often found to occur in clients with panic. Although, their causal relationship in creating panic attacks is unclear, the maintenance of PD is sustained by such catastrophic cognition as PD sufferers rely in safety seeking behavior and avoid and/or escape situations where panic occurs (130). Further support for the cognitive theory is evidenced by a causal relationship between bodily sensations and fearful cognitions (131). While the notion of nocturnal panic was once considered as being representative of support for a biological model of panic, such considerations failed to account for the selective and automatic or subliminal information processing that occurs during sleep (132–134).

CBT for PD patients promotes the development of self-efficacy which has been found to play an important part in defining the outcome of PD treatment (135). Research findings stipulate that a lack of self-efficacy in managing and dealing with PAs and misinterpretations of bodily sensations experienced during PAs are significant factors that affects treatment resistance PD (135–140). According to Barlow et al. (141), the evidence shows psychological treatments considered as more enduring after treatment termination, although pharmacological and psychological treatment have strongly demonstrated to produce equally effective treatment. Research suggests preferred strategy of PD treatment to be CBT or pharmacotherapy (11).

NICE guidelines for the treatment and management for PD includes shared decision-making and provision of information between healthcare professional and the client. This includes providing evidence-based information about PD (nature, course, treatment), discussing possible concerns around medication, taking into consideration client's prior experience with treatment, information regarding self-help groups and support groups, use of jargon-free language and accommodating to the client's language needs (translator, therapist proficient in client's preferred language) (142). These are important components to CBT as they facilitate necessary cognitive changes to impactors such as self-image, self-efficacy and literacy about panic attacks and other mental health issues.

Furthermore, as anxious individuals demonstrate deficits in inhibitory regulation and extinction learning, recent research has supported more optimal exposure strategies (16) that could easily be integrated into existing PD protocols. Inhibitory models support the linking of experiences during exposure that weaken anxiogenic associations. Moreover, the exposure facilitates a mismatch between client's expectations about their feared outcomes and their non-occurrence. Selective attention and cognitive biases also need to be targeted in successful treatment. As anxious individuals may try to distract or distance themselves from feared outcomes, therapists will typically direct attention back onto the fear during exposure and encourage consolidation of learning following the exposure. This will also augment the client's ability to retrieve any new linkages and discourage the return of the fears (16). As inhibitory associations are context-dependent, whereas excitatory associations are not (143), research has supported the need in exposure to include a variety of contextual, external or situational, internal (e.g., physiological, memory or other cognitive) and safety cues or triggers, inclusive of variability of within-exposure fear levels, in order to facilitate greater generalization and maintenance of gains. Hence, rather than relying on a behavioral habituation framework to conduct treatment, the inhibitory learning model additionally supports exposure from a planned hierarchy in a random order (16). Moreover, through its impact on expectancy violations, the inhibitory learning model supports the use of exposures where the feared outcome eventuates or is thought to eventuate. Such variations are often particularly useful in the later stages of CBT. For treatment-resistant panic disorder, the combination of CBT and pharmacotherapy is recommended as a secondary line of treatment strategy (11). When deciding the most optimal PD treatment strategy, individual characteristics of PD patients should be considered. Some patients may, for instance, be unable to tolerate the side effects of PD medications, therefore, proceeding with CBT may be beneficial whilst implicating pharmacotherapy may be better suited for patients with physical rather than cognitive symptoms of PD (11, 144–148). The development of personalized models of intervention would support a treatment formulation that incorporated individual factors guiding clinical management.

### Combination of psychological and biological factors

Some attempts have been made to compose a more integrative aetiological framework for PD. Learning theory by default integrated biological and psychological perspectives (125), whilst threat processing combined brain and mind-based theory and evidence (149). Pilecki et al. (8) scrutinized the model Fava and Morton proposed of applied causal modeling which aimed to link different components derived from biological and psychological theories (3, 8). A major criticism was that equal weight on the model was given to psychodynamic theories of PD (150), even though there is minimal empirical support in comparison to cognitive theories. On the contrary, anxiety sensitivity theory (151) is given little weight in the Fava and Morton (3) model, despite substantial evidence consistent with a causal link in the onset of PD (152). On another note, there has been little development of evidence-based approaches for combined biological-psychological treatments for PD, particularly for treatment resistant or refractory presentations (153). Multidimensional models that identify and clarify the multi-level causal mechanisms are needed to assist with the integration of biological and psychological theories and approaches (154). Kendler (155) proposed framework which integrated and synthesized information from across multiple levels of function impacted by PD and agoraphobia, demonstrates the importance of understanding of how individual risk factors and their interactions alter the outcome of a clinical phenotype (132).

## Conclusion

PD is conceptualized to be aetiologically complex, with risk determined by the interaction of multiple genetic and non-genetic risk factors spanning multiple levels of function which include biological/genetic, psychological, social, and cultural/economic (156). A summary of the theories discussed above can be seen in Table 1. The several abovementioned biological theories have been explored to explain the etiology of PD, focusing on metabolic, hormonal, genetic, neurochemical, respiratory and hyperventilation factors. Amongst the most recent approaches are the neuroscientific theories which have involved insights from neuroimaging, and translational models of animal research. Animal studies have assisted with providing added knowledge of the current fear circuitry in the brain. Whilst translating animal models of neural systems responsible for learned fear responses contributes to an understanding of PD in humans, we are far from having a satisfactory explanation of the causes and mechanisms of PD (31). Psychological theories have also been prominent, particularly cognitive behavioral models, given their superior efficacy in treatment. A number of biological and psychological factors may act as precipitating factors toward increased vulnerabilities and affects the individual's susceptibility to the development of PD, and heightened sensitivity toward and false catastrophic interpretation of psychophysiological sensations play a key role in the maintenance of the disorder. Cognitive behavioral and biological approaches that considers physiological and psychological factors interaction are likely to integrate relevant research findings that supplement of the advancement of more efficacious PD treatments. Indeed, the role of combination therapy, whereby psychological and pharmacological treatment modalities are used synergistically has been shown to have advantages in the treatment of PD (157, 158), although there may also be disadvantages particularly with respect to the impact of pharmacotherapy on developing adaptive appraisals. Three studies investigating the efficacy of CBT alone or combined CBT and pharmacotherapy found that compared to combined treatment, CBT alone produced superior results, with the studies indicating that the effects of CBT were found to be durable and long-lasting post-treatment (2, 155, 159–161). Upon treatment discontinuation, combined treatment of CBT and pharmacotherapy has been associated to greater rates of relapse (162).

**Table 1:** The summary of theories explaining the etiology of PD.

**Theory**	**Premise**	**References**
Neurochemical theory	Dysregulated functioning of neurochemicals such as deficiency in the serotonergic system or excess serotonin may be the cause of PD. Abnormal chemoreceptor reactivity may disrupt inhibitory and excitatory neurotransmitter in upregulating or downregulating panic.	(14–16)
Hormone Theory	Women are more likely to be affected by PAs. PAs rarely starts before puberty or after menopause. Pregnancy hormones appears to act as a protective factor against PAs in women.	(40–42)
Metabolic Theories	ARs located and dispersed all around the brain areas regulates the release of neurotransmitter and the action of neuromodulators, which include both inhibitory and excitatory functions. Research suggests possible implication of adenosine symptoms in panic/anxiety-inducing substances. PD sufferers are more sensitive to anxiety-inducing substances, which includes lactic acid injection, elevated CO_2_ levels, caffeine, yohimbine, m-cholorophenulpiperazine, CCK, nicotine, alcohol, epinephrine, norepinephrine, and sodium lactate.	(44–57)
Neuroanatomical Theories and Animal Model	Hypoactivation of the PFC in conditions involving intense fear observed in PD, therefore decreases the amygdala inhibition. Different DRN subdivisions were activated in PD compared to other disorders.	(28–31)
Genetic Theories	Individuals with PD may have inherited genetic predisposition toward the disorder. First-degree relative of PD patients is found to be more likely to be diagnosed with PD. Higher probability of PD diagnosis shared between monozygotic twins than dizygotic twins.	(67–72)
Respiratory Theories	Dysfunctional respiratory system may cause PAs, mainly explained by the imbalanced between O_2_ inhaled and CO_2_ exhaled during hyperventilation, thus reducing CO_2_ levels in the body triggering FSA (with dyspnea as the primary feature).	(42, 54, 78–82)
Cognitive Theories	Catastrophic thoughts and misinterpretation of somatic and other sensations after the first PA sustain the vicious cycle. An individual's low self-efficacy on panic coping abilities also contributes to the maintenance of the vicious cycle.	(6, 121–125)

Further understanding of the complex underlying mechanisms of PD needs to be supported not only by scientific insights into the biological factors but also other factors such as the genetic and epigenetic factors in relation to neurochemical, respiratory, endocrine, cognitive and behavioral systems. A multifactorial approach and model for PD may also contribute to the development of pioneering treatments which possibly targets physiological, cognitive, and behavioral symptoms of anxiety and panic.

## Author contributions

PK contributed to the conceptualization, writing, reviewing, and editing of the article. MK provided editing and review. Both authors contributed to the article and approved the submitted version.
